# Enhanced Ocular Drug Delivery of Dexamethasone Using a Chitosan-Coated Soluplus^®^-Based Mixed Micellar System

**DOI:** 10.3390/pharmaceutics16111390

**Published:** 2024-10-29

**Authors:** Samer Adwan, Faisal Al-Akayleh, Madeiha Qasmieh, Teiba Obeidi

**Affiliations:** 1 Department of Pharmaceutics and Pharmaceutical Technology, Faculty of Pharmacy, Zarqa University, Zarqa 13110, Jordan; 20160132@zu.edu.jo (M.Q.); 20199104@zu.edu.jo (T.O.); 2 Department of Pharmaceutics and Pharmaceutical Technology, Faculty of Pharmacy and Medical Sciences, Petra University, Amman 11196, Jordan; falakayleh@uop.edu.jo

**Keywords:** soluplus^®^, pluronic F-127, dexamethasone, mixed micelle, chitosan coat

## Abstract

Background: This study introduces a novel dexamethasone (DEX) mixed micellar system (DEX-MM) using Soluplus^®^ and Pluronic F-127 (PF127) to enhance ocular drug delivery. The enhancement of ocular application properties was achieved by creating a chitosan-coated DEX-MM (DEX-CMM), which promotes better adherence to the ocular surface, thereby improving drug absorption. Methods: Using the solvent evaporation method, a formulation was developed with a Soluplus^®^-to-drug ratio of 1:10, enhanced with 0.25% PF127. After dispersing in water, 1% chitosan (CS) was added. The stability and integrity of DEX within the micelles were verified using attenuated total reflection–Fourier transform infrared spectroscopy (ATR-FTIR) and differential scanning calorimetry (DSC). Additionally, in vitro and ex vivo drug release studies were conducted. Results: DEX-CMM (F6) demonstrated a particle size of 151.9 ± 1 nm and a polydispersity index (PDI) of 0.168 ± 0.003, suggesting uniformity and high electrostatic stability with a zeta potential of +35.96 ± 2.13 mV. The non-Fickian drug release mechanism indicated prolonged drug retention. Comparative analyses showed DEX-CMM outperforming a standard DEX suspension in drug release and ocular tissue permeation, with flux measurements significantly higher than the DEX suspension. Conclusion: The study confirmed the efficacy of DEX-CMM in enhancing drug delivery to ocular tissues, evidenced by improved permeability. Safety evaluations using the HET-CAM test demonstrated that DEX-CMM was non-irritant, supporting its potential for effective ocular drug delivery.

## 1. Introduction

The field of ocular drug delivery remains a pivotal focus in pharmaceutical research due to the intricate challenges associated with effectively treating eye-related conditions while maximizing therapeutic outcomes [1,2]. The eye’s complex anatomy and physiology, characterized by barriers such as the corneal epithelium, tear film, and blood–retinal barrier, pose significant obstacles to drug delivery. These barriers often result in suboptimal drug bioavailability and therapeutic efficacy, necessitating the development of advanced delivery systems capable of navigating these challenges [3,4,5].

Among the therapeutic agents used in ocular treatments, DEX holds a prominent position due to its potent anti-inflammatory and immunosuppressive properties [6,7]. DEX is extensively utilized in managing ocular inflammatory conditions, particularly in diseases affecting the posterior segment of the eye, such as diabetic macular edema (DME) and posterior uveitis [8]. These conditions are associated with severe inflammation that can lead to substantial and potentially irreversible visual impairment if not adequately managed [9]. DEX’s physicochemical limitations severely hinder its therapeutic application despite its clinical efficacy. These limitations include poor water solubility, with a solubility of approximately 10 mg/L at 25 °C and a relatively short plasma half-life ranging from 3.6 to 5.5 h [10,11]. These properties result in the need for frequent administration to maintain therapeutic drug levels, which can be burdensome for patients and increase the risk of adverse effects [12].

Addressing these challenges requires innovative drug delivery systems that can enhance the solubility, stability, and sustained release of DEX in ocular applications [13,14,15]. The emergence of nanotechnology-based delivery platforms, particularly mixed micellar systems (MMs), offers promising solutions to these limitations [16,17]. MMs, formed by the co-assembly of amphiphilic molecules in aqueous environments, have demonstrated significant potential in enhancing the solubility of poorly water-soluble drugs by encapsulating them within their hydrophobic core [18,19]. This encapsulation not only improves drug solubility but also stabilizes the drug, protecting it from degradation and facilitating its sustained release over time [20]. Moreover, the nanoscale size of MMs enables them to penetrate the ocular barriers more effectively, ensuring targeted drug delivery to the affected tissues and reducing the frequency of administration required to achieve therapeutic effects [21].

In this context, the development of a DEX-MM system represents a significant advancement in ocular drug delivery [17]. This study leverages the synergistic properties of two polymers, Soluplus^®^ and PF127, to create a robust micellar delivery system tailored for DEX. Soluplus^®^, a graft copolymer composed of polyethylene glycol (PEG), polyvinyl caprolactam (PVCL), and polyvinyl acetate (PVAc), is known for its amphiphilic nature, which allows it to form micelles that can effectively encapsulate hydrophobic drugs like DEX [22]. The hydrophilic shell of Soluplus^®^ micelles ensures stability in aqueous environments, while the hydrophobic core provides a protective environment for the encapsulated drug, thereby enhancing its solubility and bioavailability [23]. Complementing Soluplus^®^, PF127 is a non-ionic triblock copolymer that further enhances the micellar system’s functionality. PF127’s amphiphilic structure enables the formation of stable micelles that encapsulate hydrophobic drugs, enhancing their solubility and stability in ocular environments [24].

To further augment the therapeutic potential of the DEX-MM system, this study incorporates CS, a natural polysaccharide derived from chitin, into the formulation [25]. CS is well-known for its mucoadhesive properties, which significantly improve drug formulation retention time at the site of application [26]. By prolonging contact with ocular tissues, CS facilitates sustained drug release and improves DEX’s overall bioavailability in the eye [27]. Additionally, CS improves drug permeability across the corneal epithelium, which is a critical barrier to ocular drug absorption, further improving the delivery system’s therapeutic efficacy [28]. The biocompatibility and biodegradability of CS also align with the safety requirements for ocular formulations, making it an ideal component for advanced drug delivery systems [29].

The novelty of this research lies in the strategic combination of Soluplus^®^, PF127, and CS to develop MMs that address the critical therapeutic needs in ocular drug delivery. By optimizing drug encapsulation efficiency, enhancing solubility, and promoting a sustained release profile, this innovative approach offers a potential breakthrough in the treatment of ocular diseases. This system not only aims to improve the bioavailability of DEX but also seeks to reduce the frequency of administration and minimize the risk of side effects, thereby improving patient compliance and quality of life. The findings of this research have the potential to improve clinical outcomes for patients suffering from severe ocular diseases, ultimately contributing to better quality of life and reduced healthcare costs associated with these conditions.

## 2. Materials and Methods

### 2.1. Material

Low-molecular-weight chitosan (STBH6262) and Pluronic F-127 (BCCB3537) were purchased from Sigma-Aldrich^®^ (St. Louis, MO, USA). Dexamethasone (AB115220) was purchased from ABCR GmbH (Karlsruhe, Germany). Soluplus^®^ (402932-23-4) was a gift from United Pharmaceuticals (Amman, Jordan). Glacial acetic acid and ethanol was purchased from Honeywell^®^ (Raunheim, Germany). Acetonitrile HPLC grade was purchased from Sisco Research Laboratories Pvt Ltd. (Mumbai, India).

### 2.2. Methods

#### 2.2.1. Preparation of DEX-MM and DEX-CMM

The preparation of DEX-MM involved the solvent evaporation method to encapsulate DEX within mixed micelles composed of Soluplus^®^ and PF127. Soluplus^®^, PF127, and DEX were dissolved in ethanol, utilizing the minimal amount required for the complete dissolution of polymers and the drug. A 0.1% concentration of DEX, representative of the topical dose in ophthalmic formulations, was loaded at different ratios of Soluplus^®^ to PF127. Three distinct ratios of Soluplus^®^ to PF127 were utilized to explore their impact on the properties of the micellar systems. Specifically, the ratios of Soluplus^®^ to PF127 employed were 1:1, 2:1, and 4:1, respectively. The solution was subjected to vacuum drying using a precision-controlled BINDER GmbH vacuum oven (Tuttlingen, Germany), set at −0.1 MPa and 45 °C. These drying conditions were carefully chosen based on empirical data, which demonstrate their efficacy in preserving the chemical integrity and stability of pharmaceutical formulations. The drying process, lasting 24 to 48 h depending on film thickness, ensured complete solvent removal, preserving the amorphous nature of the drug and preventing recrystallization. After drying, the films were fragmented or milled into powder for further analysis. The amorphous state was confirmed by DSC, and the compatibility of the formulation components was verified using ATR-FTIR. The SD was subsequently rehydrated in 10 mL of either phosphate-buffered saline (PBS), adjusted to a pH of 7.4, or deionized water, with continuous stirring overnight at 100 rpm to ensure thorough dispersion.

**Figure 1:**
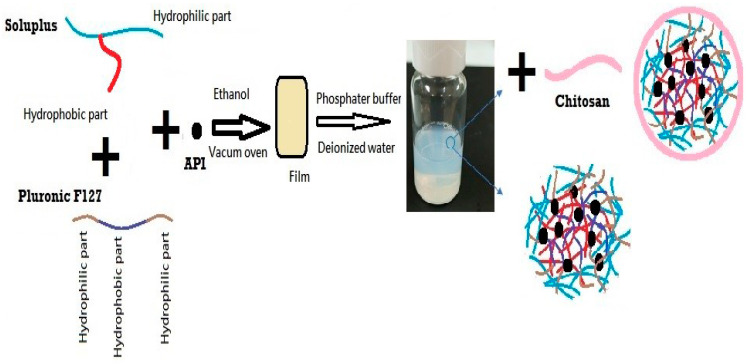
Schematic representation of the formulation process for DEX-MM and DEX-CMM.

Low-molecular-weight CS was employed for the coating process. CS was initially dissolved in a 1% *w*/*v* acetic acid solution to facilitate solubility. The concentrations of CS added varied between 0.25, 0.5, 1, and 2 mg/mL. This prepared solution was subsequently added dropwise to the micellar dispersion, ensuring a uniform coating of the micelles. Following the addition, the mixture was stirred overnight at 25 °C and 100 rpm to facilitate stable coating formation on the micelles. The final micellar formulation was filtered through a 0.22 µm membrane to ensure sterility and uniformity (Figure 1). The formulation selection was based on achieving the optimal particle size and ensuring the stability of the dispersion during storage, both of which are critical factors for effective ocular drug delivery. The composition of the selected formulations is detailed in Table 1. The content of DEX was determined by HPLC assay after diluting the micelles with acetonitrile.

#### 2.2.2. Characterization of DEX-MM and DEX-CMM

The particle size, PDI, and zeta potential of DEX-MM and DEX-CMM were measured by dynamic light scattering using a Malvern system (Malvern ZE S.A., Worcestershire, UK).

#### 2.2.3. Analytical Method

The JASCO LC-4000 Series HPLC system (Tokyo, Japan), with a binary pump, degasser, autosampler, and UV detector, was used for DEX analysis. Samples were analyzed by reversed-phase chromatography. A column from Fortis^®^ ODS-C18 (250 × 4.6 mm ID, 4 μm) RP-18 was used. The flow rate was 0.8 mL/min, and the UV detection was 245 nm. The autosampler temperature was maintained at a temperature of 25 °C. The mobile phase consisted of 60% deionized HPLC-grade water and 40% acetonitrile. The mobile phase was degassed by sonication at 37 kHz for 15 min using a Branson 1510 Ultrasonic Bath (Branson Ultrasonics Corporation, Danbury, CO, USA). The column was conditioned with the mobile phase for at least 90 min each time before use. Samples (20 μL) were injected into the HPLC system. The DEX calibration curve was made by preparing solutions containing increasing amounts of DEX (range 1–100 μg/mL) and plotting their respective UV absorption against their respective drug concentrations.

#### 2.2.4. Calculation of the Encapsulation Efficiency (%EE)

To determine the %EE of dexamethasone-loaded micelles (DEX-MM and DEX-CMM), we employed a filtration technique using Amicon Ultra centrifugal filters (15 mL, 10 kDa MWCO). This method isolated the loaded DEX from the free DEX in the formulation. After separation, samples were taken from the filtrate and analyzed using HPLC. The %EE was calculated by subtracting the amount of free DEX detected from the total amount initially loaded into the system. The result was then divided by the total DEX amount and multiplied by 100% to obtain the %EE.

#### 2.2.5. ATR-FTIR Studies

ATR-FTIR spectroscopy was conducted to assess the physical and chemical interactions among the formulation constituents, including DEX, Soluplus^®^, and PF127. The analyses were performed using a PerkinElmer^®^ Spectrum Two™ ATR-FTIR spectrometer (PerkinElmer, Llantrisant, UK). Prior to analysis, each component (polymers and DEX) was individually scanned to establish baseline spectra. For the physical mixtures, precise equimolar amounts of each component (20 mg total) were combined and ground together in a porcelain mortar for 10 min to ensure thorough mixing and intimate contact between the molecules.

To prepare the sample for ATR-FTIR analysis, the mixed powder was uniformly spread over the surface of a clean, diamond ATR crystal. This preparation method enhances the contact between the sample and the ATR crystal, which is crucial for achieving high-quality spectra. The spectral range of 4000 to 400 cm^−1^ was scanned, with a resolution of 4 cm^−1^ and 32 scans per sample to detect any shifts in characteristic peaks or new peak formations that could indicate interactions between the drug and polymer matrix.

#### 2.2.6. DSC Studies

The thermal analysis of DEX and SD was performed using a DSC 4000 differential scanning calorimeter (Perkin-Elmer, Waltham, MA, USA). Five milligrams of samples were used for DSC. Temperature scans were performed in the temperature range 25–350 °C at a heating rate of 25 °C/min under a nitrogen purge gas flow of 20 mL/min.

#### 2.2.7. In Vitro Release Study

The in vitro release behavior of DEX from the micellar formulations DEX-MM (F4) and DEX-CMM (F6) was assessed using the dialysis bag diffusion method. For comparative analysis, a free DEX suspension served as the control. Each sample consisted of 1 mL of the respective formulation, with a concentration of 1 mg/mL of DEX, and was enclosed in a dialysis membrane bag with a molecular weight cut-off of 12,000 g/mol (Green Bird Inc., Shanghai, China). These bags were then submerged in 50 mL of PBS and maintained at 37 °C to simulate physiological conditions. The completely released DEX was under the sink condition. At predetermined time intervals, triplicate aliquots of the release medium were withdrawn for analysis and immediately replenished with fresh PBS to keep the volume constant. Quantitative determination of DEX in the supernatant was carried out using HPLC. The concentration of DEX in these samples was adjusted for the volume withdrawn during sampling. Each experiment was repeated in triplicate.

#### 2.2.8. Ex Vivo Permeation Study

Sheep corneal tissues were sourced from a local slaughterhouse and were carefully selected to ensure consistency in size, thickness, and overall condition. Tissues were chosen from healthy animals of the same age group to minimize biological variability, which is crucial for maintaining uniformity in ex vivo permeation results. The eyeballs were transported in a cold PBS solution. Only the eyeballs that were transparent and had undamaged corneas were selected. Then, the corneal tissues were removed with scalpels and scissors, washed with PBS, and stored in normal saline at 4 °C until permeation study.

An ex vivo permeation study using corneal tissue was performed with Franz-diffusion cells with an effective diffusion area of 0.672 cm^2^. The receptor chamber, with a capacity of 5 mL, was filled with PBS adjusted to a pH of 7.4, and the receptor medium was stirred at 600 rpm. Corneal tissues were carefully placed on a Franz-diffusion cell, and 300 µL of F4 and F6 were applied to the donor chamber. All formulations contained 0.1 *w*/*v* % of DEX. Franz diffusion cell was maintained at 37 °C with a thermostat. A 0.5 mL sample was taken every hour from the receptor chamber for 5 h and immediately replenished with an equal volume of PBS. The permeability of the drug in each formulation was evaluated by plotting the cumulative permeated amount of DEX per unit area (µg/cm^2^) over time (h). The steady-state flux (Js) across the porcine corneal tissue was calculated from the slope of the linear portion of the cumulative permeation graph. All samples were diluted appropriately and analyzed by HPLC. The concentration of DEX in these samples was adjusted for the volume withdrawn during sampling. Each experiment was repeated in triplicate.

#### 2.2.9. Morphology Observation by Transmission Electron Microscope (TEM)

TEM imaging was performed to characterize polymeric micelles using a FEI Morgagni 268 microscope (FEI, Hillsboro, OR, USA) with an accelerating voltage of 100 kV. The samples were stained with 2% phosphotungstic acid, placed on a copper grid, and exposed under an infrared lamp for 10 min.

#### 2.2.10. Physical Stability Test

In order to evaluate the physical stability of mixed micelles, the DEX-CMM (*n* = 3) were stored at 4 °C for 15 days; then, the average size, PDI, and clarity of the micelles system were monitored. Moreover, the robustness of DEX-CMM for dilution was assessed by exposing them to 50- and 100-fold dilution with water and PBS. The diluted formulations were stored for 24 h and monitored for any physical changes (such as precipitation or phase separation).

#### 2.2.11. In Vitro Ocular Irritation Testing Using the Hen’s Egg Test–Chorioallantoic Membrane (HET-CAM)

For the HET-CAM assay, freshly fertilized hen eggs were ethically sourced from a local organic farm in accordance with institutional guidelines. These eggs were incubated at a consistent temperature of 37 °C for a period of 10 days, during which they were rotated thrice daily to prevent the embryo from adhering to one side of the shell. At the conclusion of the incubation period, the eggs were candled to identify and remove any defective ones by marking the airspace. Subsequently, the shell around the marked airspace was carefully removed using sterilized surgical blades and scissors to prepare for the assay. The inner membrane was detached using forceps to reveal the chorioallantoic membrane (CAM). F6 and controls (0.2 mL) were applied over the CAM for 5 min. The NaCl solution 0.9% *w*/*v* and NaOH solution 10% *w*/*v* were applied as controls. The test was performed in triplicate. Possible hemorrhage, vascular lysis, or the coagulation of CAM vessels were recorded for 5 min. Then, the irritation score (IS) was calculated according to a previously reported method [30].

#### 2.2.12. Statistical Analysis

Statistical analysis was performed using Student’s *t*-test or the Mann–Whitney U test, based on the data’s distribution and the experimental conditions. These statistical evaluations were carried out using GraphPad Prism software (version 6, GraphPad Software, San Diego, CA, USA). A *p*-value of less than 0.05 was deemed indicative of a statistically significant difference between comparison groups.

## 3. Results and Discussion

### 3.1. Preparation and Physicochemical Characterization

In this study, a novel formulation was developed by coating MMs of Soluplus^®^ and PF127 with CS to achieve improved stability and the ocular drug permeation of DEX. Soluplus^®^ exhibits an exceptionally low critical micelle concentration (CMC) and forms highly stable micelles against dilution [23]. In our study, the concentration of Soluplus^®^ employed exceeded the CMC (6.6 × 10^−5^), ensuring a stable dispersion. A 0.1% concentration of DEX, representative of the topical dose in ophthalmic formulations, was loaded at different ratios of Soluplus^®^ to PF127. Ethanol was utilized as a solvent for the formulation because it offers excellent solubility for both hydrophilic and hydrophobic components, such as DEX, Soluplus^®^, and Poloxamer F127 [31]. Moreover, ethanol’s advantageous properties include low toxicity and high volatility, which facilitate its complete removal by vacuum-drying at controlled temperatures, preserving the stability and integrity of the active ingredients and polymers [32]. A mixture of 100 and 2.5 mg/mL of Soluplus^®^ to PF127 resulted in stable dispersion with an optimum size for ocular drug delivery. Zhang et al. (2017) elucidated the efficacy of Soluplus^®^/PF127 MMs in augmenting the oral bioavailability of apigenin. This innovative system significantly enhanced the solubility and oral bioavailability of apigenin, underscoring the considerable potential of mixed micelles for advanced drug delivery. Leveraging a core–shell architecture, the system markedly improved the stability and solubility of hydrophobic drugs, facilitating superior absorption at targeted sites [33].

The addition of CS solution to nanoparticle dispersions is an efficient and straightforward method for CS coating and is highly advantageous in pharmaceutical applications due to its ability to enhance colloidal stability, increase bioadhesion, and control drug release [34]. CS concentrations ranging from 0.02 mg/mL to 10 mg/mL have been reported for coating various nanoparticles, with different effects on size, zeta potential, and coating efficiency [35]. In this study, two concentrations of CS, specifically 0.5 and 1 mg/mL, successfully produced a stable dispersion with an optimum particle size. The data presented in Table 2 indicate that the particle size and PDI of the selected formulations are within the optimal range for ocular drug delivery. For effective penetration through ocular barriers and to minimize irritation to the eye, nanosystems intended for this purpose should have a particle size between 10 and 200 nanometers [36]. Moreover, an ideal PDI, typically less than 0.3, is crucial as it indicates the uniform size distribution of the particles. This uniformity is essential for maintaining consistent pharmacokinetic profiles, ensuring efficient penetration through the ocular barriers, and avoiding mechanical irritation to the sensitive tissues of the eye [29]. The dispersion medium and CS concentration were primary factors influencing the particle size and zeta potential of the micelles. The size of the micelles decreased in PBS compared to those dispersed in water due to micelle contractions owing to the higher ionic strength of the medium. The addition of CS resulted in a notable increase in particle size, particularly when dispersed in water, attributed to the electrostatic attraction between the electronegative oxygen atoms in Soluplus^®^ and PF127 and the electropositive CS chain, as well as hydrogen bonding between the oxygen atoms of Soluplus^®^ and PF127 and hydroxyl groups of CS.

The addition of CS shifted the zeta potential toward more positive values, with a greater effect observed in water than in PBS. The zeta potential of DEX-MM was shifted from −3.17 ± 0.74 to −0.59 ± 1.34 and 3.20 ± 1.87 by the addition of 0.5 mg/mL and 1 mg/mL CS, respectively, and dispersion in PBS. By contrast, the charge was shifted from −4.37 ± 0.57 to 0.26 ± 0.36 and 35.96 ± 2.13 by the addition of 0.5 mg/mL and 1 mg/mL CS, respectively, and dispersion in the water. Pepic et al. (2008) provided insights into how different parameters influence the properties of CS and surfactant-based nanoparticle systems. They reported that higher ionic strength in the dispersion medium can diminish electrostatic repulsions by shielding charges on CS and surfactant molecules, leading to larger particle sizes due to aggregation and a reduction in zeta potential by compressing the electrical double layer around the particles [37]. Additionally, higher concentrations of CS result in larger particle sizes due to increased viscosity and the formation of a thicker polymer layer, although excessively high concentrations may cause agglomeration. Conversely, the zeta potential increases with the CS concentration, enhancing electrostatic stability and preventing aggregation [38]. At acidic pH levels, CS is more soluble, leading to smaller particles and higher zeta potential due to increased protonation, whereas, at a higher pH, the decreased solubility and protonation of CS lead to larger particles and lower zeta potential [38]. Micelles with higher zeta potentials typically exhibit greater electrostatic repulsion between particles, resulting in improved stability against aggregation and precipitation [39]. This enhanced stability ensures prolonged circulation time in biological fluids, facilitating targeted drug delivery to specific sites in the body.

The positive zeta potential is a key factor enhancing the interaction between the micelles and biological membranes, especially considering that the ocular surface and most cell membranes are negatively charged due to the presence of sialic acids and phospholipids. The positive zeta potential of micelles not only contributes to electrostatic interactions with negatively charged cell membranes but also promotes adhesion to mucosal surfaces, thereby increasing retention time at the site of the application. This is particularly valuable in ocular drug delivery, where drugs are often cleared rapidly due to blinking and tear fluid turnover [40]. By prolonging the contact time with the corneal and conjunctival tissues, the positively charged micelles ensure that a higher concentration of the drug is available for absorption over an extended period. Moreover, the positive zeta potential enhances cellular uptake via adsorptive endocytosis, a process driven by electrostatic interactions between the cationic micelles and the anionic cell membranes. This facilitates the internalization of micelles into cells, allowing for more effective intracellular drug delivery [41]. For poorly water-soluble drugs such as dexamethasone, this property is particularly advantageous as it enhances drug bioavailability by overcoming the limitations posed by biological barriers, such as the tight junctions of the corneal epithelium.

### 3.2. ATR-FTIR Studies

**Figure 2:**
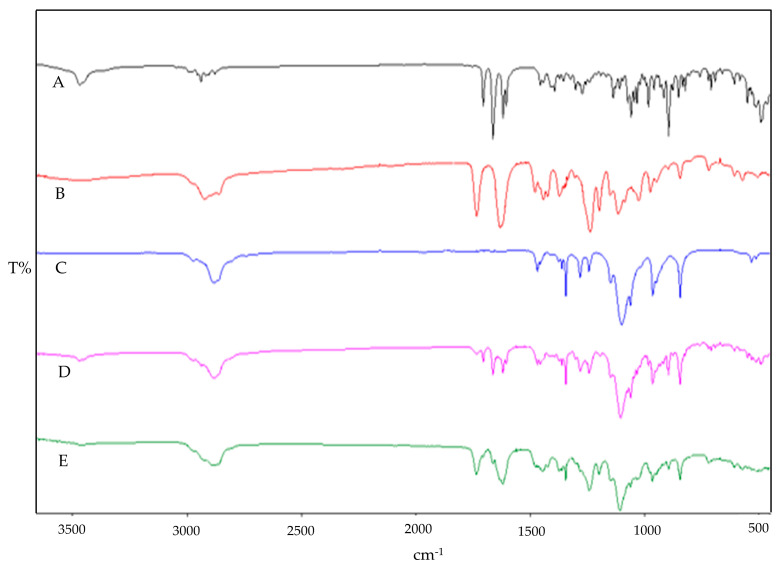
FTIR fingerprint of individual components, physical blend, and SD: DEX (A), Soluplus^®^ (B), PF127 (C), physical mixture (D), and (E) SD.

Figure 2 illustrates the distinctive spectral features observed in the infrared spectra of pure DEX, Soluplus^®^ and PF127. In the DEX spectrum, characteristic absorption bands were detected at 3390 cm^−1^ and 1268 cm^−1^ and attributed to the stretching vibrations of O-H and C-F bonds, respectively. Additionally, peaks at 1706 cm^−1^, 1662 cm^−1^, and 1621 cm^−1^ indicated the presence of C-O and a double bond framework conjugated to C-O bonds [42]. The Soluplus^®^ spectrum displayed characteristic bands at 1730 cm^−1^ (corresponding to ester carbonyl stretching) and 1632 cm^−1^ (representing tertiary amide carbonyl), consistent with previous studies [43]. Furthermore, the PF127 spectrum exhibited signals associated with the stretching vibrations of various bonds: 2880 cm^−1^ (C–H stretch), 1278 cm^−1^, and 1240 cm^−1^ (C–O–C stretches), and 1097 cm^−1^ (C–O stretch) [44]. Upon examining the physical mixture and the SD, it was evident that while the characteristic signals of DEX and the polymers were present in the spectra of the physical mix, SD displayed a reduction in the intensity of DEX absorption peaks. This reduction suggests the occurrence of intermolecular interactions and the entrapment of DEX within the SD formulation [45]. This observation underscores the potential role of intermolecular interactions in the formation and stability of DEX-loaded micelles, which could influence their physicochemical properties. These findings contribute to our understanding of the structural characteristics of the developed formulations and their potential applications in drug delivery systems.

### 3.3. DSC Studies

**Figure 3:**
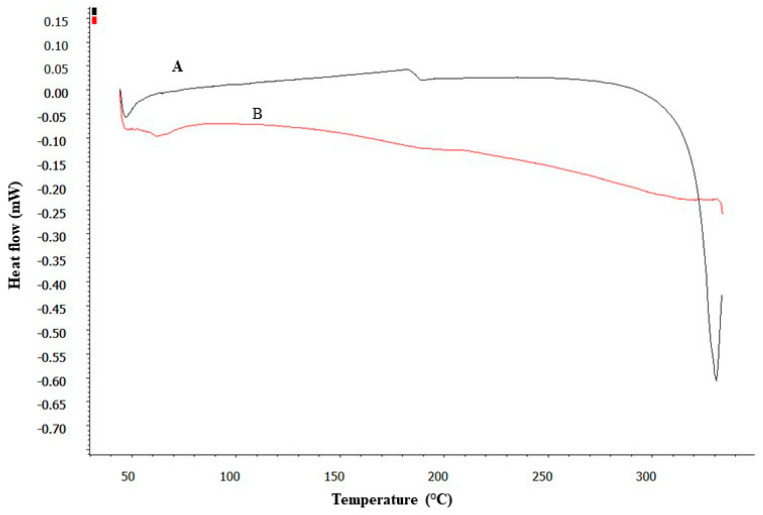
DSC thermograms of pure DEX (A) and the SD (B).

DSC analysis provided insightful data on the thermal properties of pure DEX and its formulation (SD). As shown in Figure 3, the DSC thermogram of pure DEX displayed a distinct endothermic peak, suggesting a melting point ranging between 280 and 290 °C. This peak is characteristic of the crystalline nature of pure DEX [46]. In contrast, the DSC thermogram of the SD showed a significant alteration; the characteristic melting peak of DEX was absent. This absence indicated a transformation in the physical state of DEX within the micelles from crystalline to amorphous [46]. Such a transformation is indicative of the effective encapsulation of DEX within the micelle matrix, where the drug is molecularly dispersed and no longer exists in its crystalline form.

This finding is crucial as it suggests that the drug is well-integrated into the micelles, potentially enhancing its solubility and stability. Thus, the development of DEX-CMM could represent a significant advancement in the formulation of DEX for enhanced drug delivery.

### 3.4. In Vitro Drug Release

**Figure 4:**
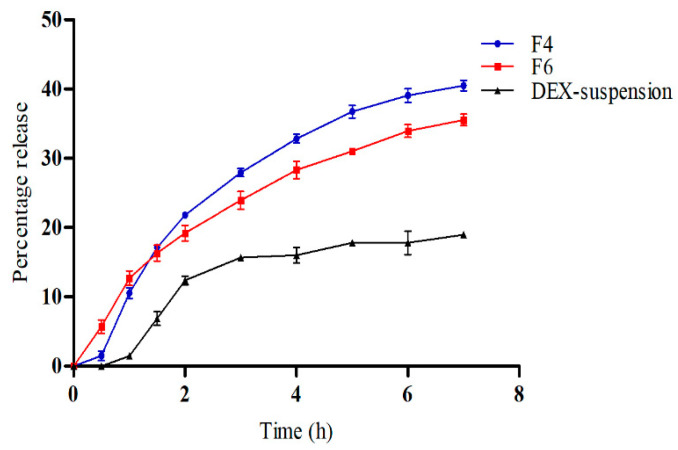
The in vitro drug release results. Plotted in terms of % of released DEX vs. time for DEX suspension, DEX-MM, and DEX-CMM (Mean ± SD, *n* = 3).

Figure 4 illustrates the in vitro release profiles of DEX from DEX suspension, F4 (DEX-MM), and F6 (DEX-CMM) in PBS. The results reveal a notable improvement in drug release from both DEX-MM (*p* = 0.0419) and DEX-CMM (*p* = 0.0373) when compared to the DEX suspension. This augmented release can be attributed to the solubilizing effect of Soluplus^®^ and PF127 on the drug. After 1 h, DEX exhibited a percentage release of 10.57 ± 0.79% and 12.70 ± 1.06% from formulations F4 and F6, respectively, while the released amounts after 7 h were 35.53 ± 0.83% and 40.50 ± 0.75% from F4 and F6, respectively. Statistical analysis indicated a significant difference (*p* = 0.0338) in drug release rates between formulations F4 and F6, underscoring the substantial impact of the chitosan coating on the release dynamics compared to the non-coated DEX-MM system.

In this study, several kinetic models were evaluated to describe the release of dexamethasone (DEX) from the DEX-CMM system, including the first-order, zero-order, Higuchi, and Korsmeyer–Peppas models. Each model provides insights into the release behavior but also presents limitations in its application. The first-order model assumes that drug release is proportional to the remaining drug concentration in the system, which is applicable for soluble drugs. However, this model does not fully account for systems where release is influenced by both diffusion and the degradation of the polymer matrix. In this study, the first-order model yielded a relatively low R^2^ value (0.7592), indicating that it was not the best fit for describing the DEX-CMM system. The zero-order model, which assumes a constant release rate independent of drug concentration, is often ideal for controlled release systems. However, it is less suitable for systems where release mechanisms involve both diffusion and erosion. In our analysis, the R^2^ value for the zero-order model was 0.9350, suggesting a better fit than the first-order model, but this was still insufficient for capturing the complexities of the DEX-CMM release mechanism. The Higuchi model, based on Fickian diffusion, is commonly used to describe drug release from matrix systems. This model assumes that diffusion through a homogeneous polymer matrix controls drug release [47]. The high R^2^ value of 0.9624 in our study indicates that diffusion plays a major role in the release of DEX from the DEX-CMM system. However, the Higuchi model does not account for polymer erosion, which is likely to influence release in CS-coated micelles. The Korsmeyer–Peppas model, which also exhibited a high R^2^ of 0.9555 in our study, provides further insight into the complexities of the release mechanism. The release exponent of 0.65 indicates anomalous (non-Fickian) diffusion, where both the diffusion of the drug and the erosion of the polymeric matrix contribute to the release process [48]. This dual mechanism is particularly pertinent to CS-coated systems, where the polymer may undergo swelling or erosion, thereby influencing the release dynamics [49]. Additionally, the CS coating modifies the release behavior by adding a physical barrier that the drug molecules must traverse, further affecting the release kinetics [50].

### 3.5. Ex Vivo Drug Permeation

**Figure 5:**
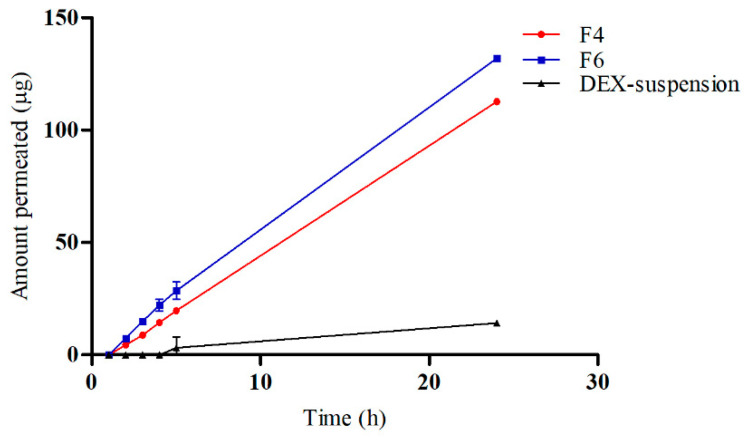
Ex vivo permeation study results of DEX suspension, DEX-MM, and DEX-CMM through corneal tissue using the Franz cell (Mean ± SD, *n* = 3).

To evaluate the permeation and penetration capabilities of DEX, sheep corneal tissues were employed as an ex vivo model (Figure 5). The study compared three formulations: F4, F6, and a DEX suspension. The results indicated that DEX permeated the corneal tissues from both F4 and F6 with a lag time of 1.5 h. In contrast, the DEX suspension exhibited a significantly delayed permeation, with drug permeation commencing only after 4 h. The flux (Js) values were calculated to quantify the permeation rates. The DEX suspension displayed a flux of 0.65 ± 0.05 mg/cm^2^/h, which was substantially lower compared to the F4 and F6, which showed flux values of 4.92 ± 0.02 mg/cm^2^/h and 5.63 ± 0.13 mg/cm^2^/h, respectively. These findings highlight the superior permeation capabilities of micellar formulations over the traditional suspension. The lag time of 1.5 h for the micellar formulations suggests a rapid initial uptake of DEX through the corneal barrier, which is likely facilitated by the enhanced solubility and stability provided by the micelles. In contrast, the prolonged lag time for the DEX suspension underscores the challenges of delivering hydrophobic drugs across the corneal barrier using conventional suspension formulations. The significantly higher flux values for CS-coated micelles (*p* = 0.0397), representing a 766% increase in F6 compared to the control DEX suspension, highlight the beneficial impact of CS in enhancing drug permeation. CS can temporarily disrupt tight junctions between corneal epithelial cells, facilitating paracellular drug transport and enhancing drug permeation across the cornea. Furthermore, CS’s positive charge allows for electrostatic interactions with negatively charged mucin glycoproteins present on the ocular surface, leading to increased residence time and improved drug absorption [51].

### 3.6. TEM Characterization of Nanoparticle Morphology

**Figure 6:**
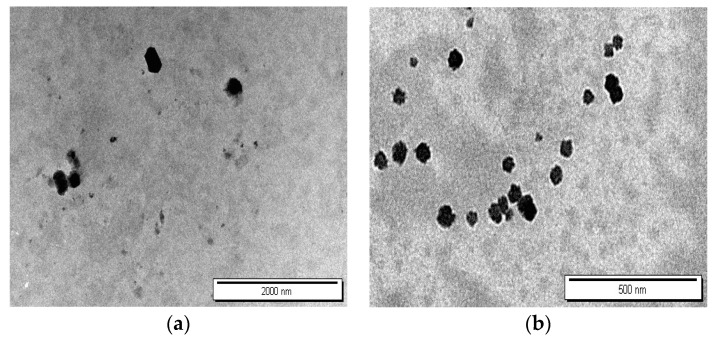
TEM images of F6 (**a**) and F4 (**b**).

The morphological characteristics of formulations F4 and F6, corresponding to DEX-MM and DEX-CMM, respectively, were meticulously analyzed using TEM. The TEM images, displayed in Figure 6, illustrate the distinct morphologies of DEX-MM and DEX-CMM under various magnifications. Figure 5 highlights that DEX-MM and DEX-CMM micelles are predominantly round in shape and exhibit minimal aggregation. Notably, the surface-modified nanomicelles (DEX-CMM) are more compact and have a larger diameter. This is speculated to result from the micelles being coated with multiple entangled CS molecular chains, which enhances their structural integrity [52]. The CS enhancement on the surface of DEX-CMM offers improved stability and increased residence time on ocular surfaces, making them highly suitable for use as carriers in ophthalmic drug delivery systems [53].

### 3.7. Physical Stability of DEX-CMM

The physical stability of F6 was assessed after storage at 4 °C. The data presented in Table 3 indicate that both the average particle size and the PDI remained stable, showing no significant changes after a 15-day storage period at this temperature (*p* = 0.3927). Additionally, the formulation remained clear throughout the storage period, showing no signs of aggregation or precipitation. This stability underscores the robustness of the formulation under refrigerated conditions, highlighting its potential for sustained use in clinical settings [54]. Additionally, F6 was exposed to different folds of dilution in PBS to mimic in vivo conditions where the formulation would encounter gradual dilution. The formulation was subjected to a 50- and 100-fold dilution in PBS [55]. This formulation showed no signs of precipitation, cloudiness, or separation for 24 h.

### 3.8. In Vitro Ocular Irritation Testing Using HET-CAM

**Figure 7:**
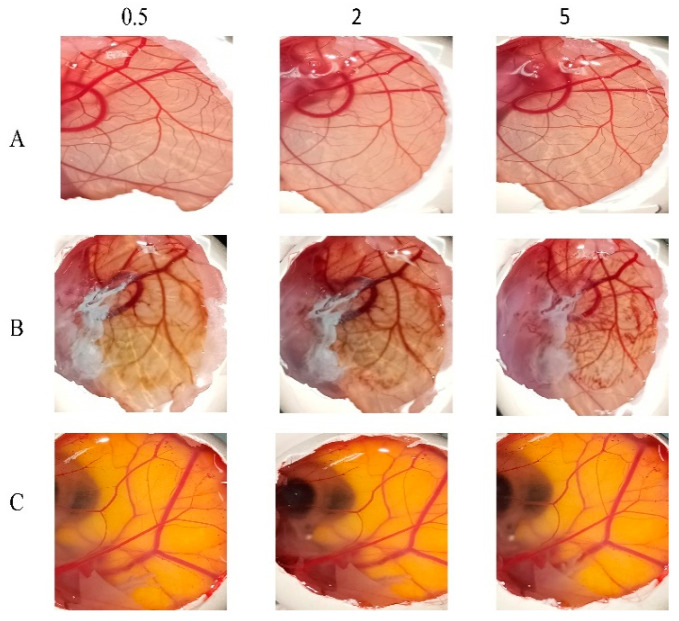
HET-CAM analysis depicting the response to F6 (**A**), 10% *w*/*v* NaOH as a positive control (**B**), and normal saline solution as a negative control (**C**). Images captured at 0.5, 2-, and 5-min post-application illustrate the comparative irritancy and biocompatibility of the formulations.

The potential irritancy of substances on the eye was studied in vitro using HET-CAM. Applying HET-CAM offers many advantages, one of which is the well-developed vascularization of the CAM as tissue-containing arteries, veins, and capillaries, which responds well to injury with an inflammatory reaction similar to that observed in rabbit conjunctival tissues. The second advantage is the reduced cost (due to the reduction in the number of mammals being used), as well as the time and suffering of the mammals [56,57]. As presented in Figure 7, it is apparent that the positive control (10% NaOH) was severely irritant, in contrast with the negative controls (aqueous; 0.9% NaCl) in producing a non-irritant response. F6 was found to be practically non-irritant when applied to the surface of the CAM because no hemorrhage, vascular lyses, or coagulation of CAM vessels was observed during the 5 min of the test.

## 4. Conclusions

This study heralds a significant advancement in ocular drug delivery by developing and thoroughly characterizing DEX-CMM. By incorporating Soluplus^®^ and PF127, the micelles enhance the solubility and stability of DEX well beyond its critical micelle concentration, resulting in a stable and efficacious dispersion suitable for ocular application. This addresses common issues with conventional DEX treatments, which often suffer from poor solubility and instability. Additionally, the integration of CS significantly boosts the mucoadhesive properties of the system, leading to extended drug retention on the ocular surface and improved permeation through the ocular barriers. This is particularly beneficial for treating diseases affecting the posterior segment of the eye, where traditional methods frequently fall short. The study also establishes how DEX-CMM achieves an optimal particle size and zeta potential, which are crucial for maximizing bioavailability and ensuring stable, effective, and irritation-free delivery to targeted ocular tissues. These characteristics underline the potential of DEX-CMM to enhance therapeutic outcomes significantly.

Looking forward, it is crucial to explore the long-term stability of DEX-CMM under various storage conditions to ensure its efficacy over extended periods. Additionally, in vivo studies are essential to assess the pharmacodynamics, pharmacokinetics, and long-term safety of DEX-CMM, providing deeper insights into its clinical relevance and therapeutic potential. Addressing the scalability challenges of this innovative formulation will also be vital for its successful transition from laboratory to clinical use. Further research should also expand on the bioefficacy of DEX-CMM across different ocular conditions to fully understand its therapeutic versatility and potential.

In conclusion, this study represents a significant advancement in pharmaceutical sciences by developing an advanced ocular drug delivery system. It demonstrates the potential to enhance treatment protocols and improve patient outcomes in eye care. The findings support the further research and refinement of micellar systems, particularly those enhanced with biocompatible materials, such as chitosan, which aim to address the persistent challenges in managing ocular diseases effectively. Soluplus^®^-PF127-based micelles, augmented with a chitosan coating, show considerable promise for delivering a broad spectrum of therapeutic agents. While this research centered on the ocular delivery of DEX, the versatility of this system allows for its application to other poorly water-soluble drugs, including corticosteroids, anticancer agents, and antiviral medications. These micelles enhance drug solubility and stability, facilitate controlled release, and improve penetration through biological barriers. Additionally, their mucoadhesive properties make them suitable for non-ocular applications, such as nasal or oral drug delivery, where enhanced absorption and prolonged retention are crucial. This study underscores the potential of this micellar system as a versatile platform for drug delivery across various therapeutic areas.

## Figures and Tables

**Table 1:** Composition of selected formulations. Concentrations expressed in mg/mL.

Formulation	Soluplus^®^	PF127	CS	DEX	Medium
F1	10	25	0	1	PBS
F2	10	25	0.5	1	PBS
F3	10	25	1	1	PBS
F4	10	25	0	1	DDW
F5	10	25	0.5	1	DDW
F6	10	25	1	1	DDW

**Table 2:** Characterization of DEX-MM and DEX-CMM formulations (Mean ± SD, *n* = 3).

Code	Size (nm)	PDI	Zeta Potential (mV)	%EE
F1	76.9 ± 0.8	0.160 ± 0.004	−3.17 ± 0.74	81.32 ± 1.96
F2	92.2 ± 0.2	0.139 ± 0.007	−0.59 ± 1.34	75.62 ± 2.93
F3	107 ± 0.1	0.125 ± 0.007	3.20 ± 1.87	76.90 ± 1.02
F4	69.1 ± 0.6	0.053 ± 0.007	−4.37 ± 0.57	89.44 ± 1.37
F5	118.5 ± 1.5	0.157 ± 0.004	0.26 ± 0.36	85.50 ± 1.90
F6	151.9 ± 1	0.168 ± 0.003	35.96 ± 2.13	91.95 ± 0.46

**Table 3:** Physical stability of F6 over time at 4 °C (Mean ± SD, *n* = 3).

Time (d)	1	3	7	10	15
Size (nm)	151.9 ± 1	148.4 ± 1.2	151.4 ± 1.7	149.2 ± 0.5	145.9 ± 3.1
PDI	0.168 ± 0.003	0.166 ± 0.001	0.187 ± 0.007	0.182 ± 0.008	0.197 ± 0.011

## Data Availability

The data that support the findings of this study are available from the corresponding author (Samer Adwan).
